# Host-Associated *Bacteroides* 16S rDNA-Based Markers for Source Tracking of Fecal Pollution in Laguna Lake, Philippines

**DOI:** 10.3390/microorganisms11051142

**Published:** 2023-04-27

**Authors:** Gicelle T. Malajacan, Mae Ashley G. Nacario, Marie Christine M. Obusan, Windell L. Rivera

**Affiliations:** 1 Pathogen-Host-Environment Interactions Research Laboratory, Institute of Biology, College of Science, University of the Philippines Diliman, Quezon City 1101, Philippines; gtmalajacan@up.edu.ph (G.T.M.); mgnacario@up.edu.ph (M.A.G.N.); 2 Microbial Ecology of Terrestrial and Aquatic Systems Laboratory, Institute of Biology, College of Science, University of the Philippines Diliman, Quezon City 1101, Philippines; mmobusan@up.edu.ph

**Keywords:** 16S rDNA, *Bacteroides*, fecal pollution, microbial source tracking (MST), Laguna Lake, Philippines, library-independent MST

## Abstract

Sources of fecal contamination in Laguna Lake, Philippines, were identified using a library-independent microbial source tracking method targeting host-associated *Bacteroides* 16S rDNA-based markers. Water samples from nine lake stations were assessed for the presence of the fecal markers HF183 (human), BoBac (cattle), Pig-2-Bac (swine), and DuckBac (duck) from August 2019 to January 2020. HF183 (average concentration = 1.91 log_10_ copies/mL) was the most frequently detected, while Pig-2-Bac (average concentration = 2.47 log_10_ copies/mL) was the most abundant. The detected marker concentrations in different stations corresponded to the land use patterns around the lake. Generally, all marker concentrations were higher during the wet season (August–October), suggesting the effect of rainfall-associated factors on the movement and retention of markers from sources. There was a significant association (ρ = 0.45; *p* < 0.001) between phosphate and the concentration of HF183, suggesting domestic sewage-derived pollution. The markers had acceptable sensitivity and specificity, i.e., HF183 (S = 0.88; R = 0.99), Pig-2-Bac (S = 1.00; R = 1.00), and DuckBac (S = 0.94; R = 1.00), and therefore may be used for the continuous monitoring of fecal pollution in the lake and in designing interventions to improve the quality of the lake water.

## 1. Introduction

Laguna Lake, the largest inland water body in the Philippines, serves various domestic, agricultural, and industrial purposes for an estimated 14.6 million users in the region [1,2]. However, the rapid increase in population and urbanization in the Laguna Lake area in recent years has contributed to the decline in the quality of the lake water [3,4]. Increased fecal coliform from different point and non-point sources of contamination in the water body and its tributaries exposes the public to risks associated with waterborne diseases. Point sources are pollution sources that are commonly discharged in water bodies that can be directly identified, while non-point sources are the cumulative contaminations from different sources that diffuse into the environment [5]. In addition to presenting serious health risks, fecal pollution has a negative impact on economic activities and can lead to environmental degradation [6]. Therefore, it is necessary to monitor the levels of fecal pollution in the lake and to determine its point sources.

As part of water quality monitoring, microbiological parameters are evaluated to determine the extent of fecal pollution. Fecal indicator bacteria (FIB), such as *Escherichia coli* and other enterococci, are currently used to assess the level of microbiological pollution in fresh and coastal waters. However, these microorganisms are present in both human and animal feces [7], and so their detection does not discriminate the origin of the fecal pollution [7]. Microbial source tracking (MST) methods have been developed to distinguish between contamination from human and animal sources such as cattle, swine, poultry, etc. Library-independent MST uses host-specific microbial indicators to accurately track fecal sources. Bernhard and Field [8] suggested the use of *Bacteroides* species as indicators of fecal pollution due to their abundance in the feces of warm-blooded animals. *Bacteroides* are strict anaerobes and do not survive for long in aerobic conditions. Therefore, their presence in environmental waters suggests continuous fecal inputs, making *Bacteroides* an ideal marker of fecal pollution. Several assays have been designed to target specific sequences within the *Bacteroides* 16S rRNA gene, and to differentiate human-derived contamination from that of other animals. Markers for the estimation of fecal pollution from pigs [6], dogs [9], humans [10,11], cows [10,11], and poultry [12] have been designed and evaluated. However, due to their variability in validity and efficiency when applied in different regions, there are no gold standards for human and nonhuman markers that are applicable to all geographical areas [13,14]. 

Sensitivity and specificity are critical parameters for determining the efficiency and validity of MST markers [15]. These criteria refer to the proportion of true positives (sensitivity) and true negatives (specificity) in the population. *Bacteroides* 16S rDNA-based markers for humans, cattle, swine, and poultry have been evaluated based on host-specificity and host-sensitivity in various geographical areas [16,17,18,19,20]. Among these markers, HF183 [10], BoBac [11], Pig-2-Bac [6], and DuckBac [12] achieved the recommended value (≥0.80) for a reliable and effective marker for both criteria [6,14]. ChickenBac has relatively weak sensitivity (0.69) but good specificity (0.92) [12]. However, it is important to know whether a particular marker would function differently in the Philippine setting, specifically in Laguna Lake.

In this study, human and animal sources of fecal contamination in Laguna Lake were investigated using the *Bacteroides* 16S rDNA-based markers HF183, BoBac, Pig-2-Bac, and DuckBac. The specific objectives were to (1) validate the performance of *Bacteroides* 16S rDNA markers specific for each host, (2) quantify and determine the key sources of pollution in the lake, (3) assess the spatiotemporal variations in marker abundance across sampling seasons and stations, and (4) investigate any correlations between water quality parameters and the presence of markers.

## 2. Materials and Methods

**Sample collection and processing of lake water samples**. Fresh feces from five host sources, namely, humans (*n* = 24), cows (*n* = 20), pigs (*n* = 15), ducks (*n* = 18), and chickens (*n* = 23), were collected from different farms and municipalities of Metro Manila, Laguna, and Rizal, sites which are located around Laguna Lake, Philippines. Research ethics clearance was obtained from the University of the Philippines Manila Research Ethics Board (UPMREB Code: 2018-356-01).

**Figure 1:**
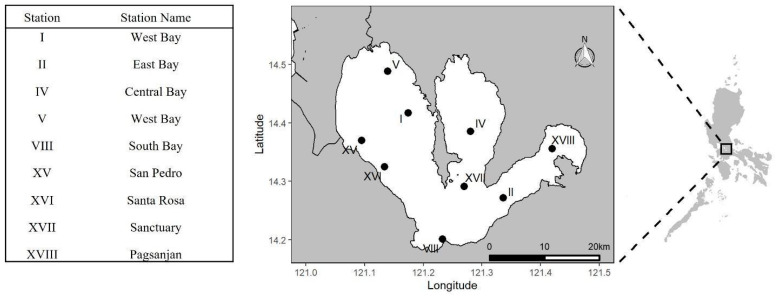
**Map of Laguna Lake sampling stations (generated through R programming).** Nine sampling stations were selected to adequately represent each part of the lake.

One liter of water from the selected Laguna Lake stations (Figure 1) was collected monthly from August 2019 to January 2020, with August–October and November–January representing the wet and dry seasons, respectively [21]. All fecal and water samples were transported on ice and processed immediately in the laboratory.

Environmental DNA was harvested by filtering 1 L of each water sample through a 0.45 µM membrane (Pall Corporation, Ann Arbor, MI, USA). The membrane filter was then resuspended in 5 mL of 1× TE (10 mM Tris-HCl and 1 mM EDTA) buffer or sterile type I water and vortexed vigorously to capture the adhered microorganisms. The final suspension was centrifuged at 14,000× *g* for 10 min to separate the pellet from the buffer. All pellets were stored at −20 °C until DNA extraction.

**Measurement of water quality parameters.** The measurements of the following water quality parameters, monitored from August 2019 to January 2020, were obtained from the Laguna Lake Development Authority (LLDA): pH (units), ammonia (mg/L), nitrate (mg/L), inorganic phosphates (mg/L), biological oxygen demand (BOD, mg/L), dissolved oxygen (DO, mg/L), and fecal coliform count (MPN/100). 

**DNA extraction from fecal and lake water samples.** DNA extraction was performed using a PureLink™ Microbiome DNA Purification Kit (Invitrogen, Thermo Fisher Scientific, Carlsbad, CA, USA). Two hundred milligrams of fecal sample were used as the starting material, and extraction was performed by following the manufacturer’s protocol (Pub. No. MAN0014266). For water samples, the pellet was used as the starting material for the extraction of DNA, following the manufacturer’s protocol (Pub. No. MAN0014322). The final eluted DNA (100 µL) was then stored at −20 °C until further use.

**Validation of host-associated *Bacteroides* markers.** PCR-based assays were performed to determine the efficiency of candidate host-specific markers from published studies (Table 1) [6,10,11,12]. The performance criteria were host-specificity (S), host-sensitivity (R), and accuracy (A) for the detection of human-, swine-, cattle-, and poultry-specific contamination in Laguna Lake. Briefly, 10 µL of GoTaq Green Master Mix (Promega Corporation, Fitchburg, WI, USA), 1.0 µL of 10 µM forward and reverse primers, 2 µL of DNA, and nuclease free water, for a total of 20 µL per reaction, were mixed in a 200 µL tube. In a thermal cycler, the conditions were set at 95 °C for 5 min for initial denaturation, 30 cycles at 95 °C for 15 s, different annealing temperatures for each primer for 30 s, and 72 °C for 1 min. Final extension was performed at 72 °C for 5 min, and a holding temperature of 12 °C was set infinitely. To confirm the presence of a single product of the correct size, amplicons were subjected to 1.5% agarose gel electrophoresis and visualized under a UV Gel Documentation System (Bio-print ST4, Vilber Lourmat, Marne La Vallee, France).

The number of positive and negative results were used to calculate the metrics for marker performance following Kildare [22]: (1) true positives (TP) indicated the number of target host samples that tested positive for the assayed marker, (2) true negatives (TN) indicated the number of nontarget samples that tested negative for the assayed marker, (3) false positives (FP) indicated the presence of the marker in nontarget hosts, and (4) false negatives (FN) indicated the absence of the marker in the target host. The equations provided by Somnark [23] are as follows:

R = TP/TP + FN(1)

S = TN/TN + FP(2)

A = (TP + TN)/(TP + TN + FP + FN)(3)

where R represents host-sensitivity, S represents host-specificity, and A represents accuracy.

**Detection and quantification of host-associated contamination in lake waters.** The reaction mix was prepared by mixing 10 µL of SensiFAST™ SYBR^®^ Hi-ROX (Bioline, UK), 6 µL of nuclease-free water, 0.8 µL of each primer, and 2 µL of the sample or standard. The standard used was optimized from amplicons of markers known to be specific to *Bacteroides*. All reactions were performed in duplicate along with the no-template control. The cycling parameters for qPCR (StepOnePlus™ Real-time PCR system; Applied Biosystems, Woburn, MA, USA) were as follows: initial denaturation at 95 °C for 2 min, 40 cycles at 95 °C for 5 s, 60°C for 10 s, and 72 °C for 10 s. Melting curve analyses were set by default with the following conditions: 1.6 °C/s, 95 °C for 15 s, 1.6 °C/s, 60 °C for 1 min, and 0.15 °C/s, 95 °C for 15 s (Applied Biosystems, Waltham, MA, USA). The resulting standard curve was analyzed to assess the linearity and efficiency of amplification, and the dissociation curve was evaluated to determine the presence or absence of nonspecific amplifications. The resulting DNA marker concentrations were converted into DNA copy numbers using the standard formula given below:

(4) $$\mathrm{number}\;\mathrm{of}\;\mathrm{copies}\;\left(\mathrm{molecules}\right)=\frac{X\;\mathrm{ng}\;\times 6.0221\times 10^{23}\;\mathrm{molecules}/\mathrm{mole}}{\left(N\;\times 660\frac{g}{\mathrm{mole}}\right)\times 1\times 10^{9}\;\mathrm{ng}/g}$$

where X represents the DNA concentration and N represents the number of marker base pairs. 

**Analysis of data.** Data were analyzed using R v.4.0.4 [24]. The concentrations of *Bacteroides* markers (copy number mL^−1^) were initially log_10_-transformed, and those that were not detected were assumed to be below the limit of detection (<LOD); these were then used to assess the spatiotemporal patterns of fecal contamination in the lake. The percentage of occurrence for each marker was calculated by dividing the number of times the marker was detected by the number of sampling events (*n* = 6) throughout the duration of the experiment. The association between the *Bacteroides* markers and water parameters was determined using the nonparametric Spearman correlation. Single imputation was performed for <LOD observations by dividing the LOD of each marker by 2 [25]. The resulting *p*-values were adjusted for multiple comparisons using Holm’s correction [26].

## 3. Results

### 3.1. Validation of Bacteroides 16S rDNA-Based Markers for MST

A total of 100 fecal samples was used to validate the performance characteristics of the five host-associated markers, namely, HF183, BoBac, Pig-2-Bac, DuckBac, and ChickenBac (Table 2). HF183 was only detected in 5% of the chicken fecal samples, while BoBac was present in 80–100% of the nontarget host samples. Neither the Pig-2-Bac or DuckBac markers were found in any of the nontarget host samples. Lastly, ChickenBac was present in 75–100% of the nontarget host samples, but not in the human samples.

Among the five markers, Pig-2-Bac, DuckBac, and HF183 achieved the recommended value (≥0.80) for both sensitivity and specificity (Table 3). Pig-2-Bac performed best (S = 1.00; R = 1.00), followed by DuckBac (S = 0.94; R = 1.00), and HF183 (S = 0.88; R = 0.99). By contrast, ChickenBac (S = 0.65; R = 0.31) and BoBac (S = 0.90; R = 0.10) performed poorly, and hence these two markers were not used further for source tracking analysis. 

### 3.2. Detection and Quantification of Host-Associated Contamination in Lake Waters

**Figure 2:**
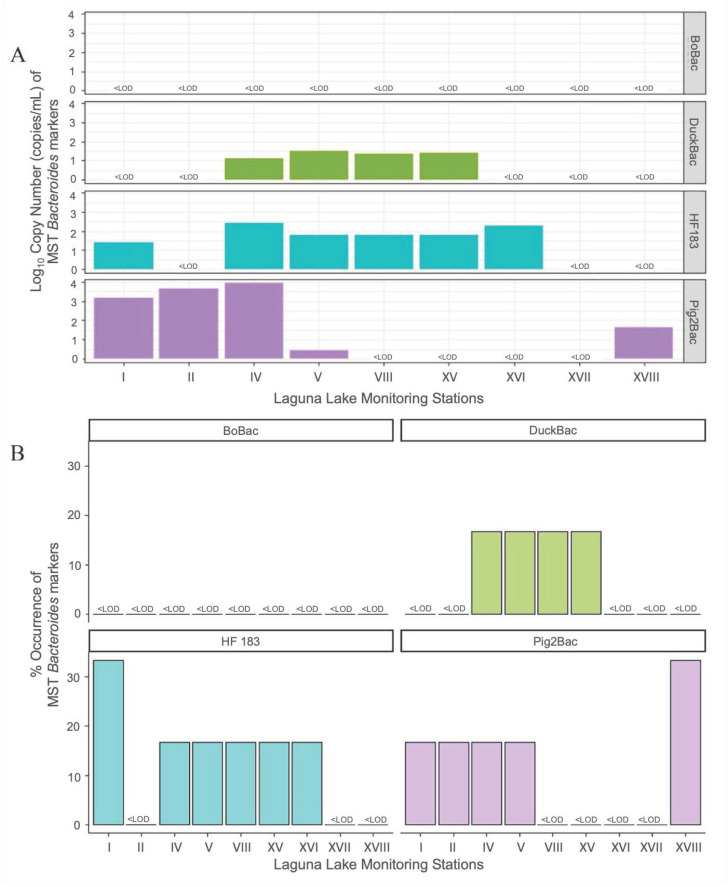
**Log copy number (copies mL^−1^, (A)) and occurrence (%, (B)) of *Bacteroides* markers in the Laguna Lake monitoring stations from August 2019 to January 2020.** Those that were below the limit of detection are shown as <LOD. qPCR analysis was carried out to detect and quantify host-associated contamination in water samples collected from nine sampling sites. The resulting copy numbers from each sampling site for each source were averaged to determine the most abundant source of contamination in the lake. The occurrence of each source was also obtained by measuring the frequency of detection of each marker across all nine sampling sites within the given sampling period.

The qPCR analysis of a total of 54 water samples using the host-associated markers revealed the most abundant contributors of fecal contamination in selected Laguna Lake stations from August 2019 to January 2020 (Figure 2). Pig-2-Bac was the most abundant and had the highest mean concentration (2.47 log_10_ copies/mL), which was detected in five out of nine stations. Pig fecal contamination was most abundant in the Central Bay (Station IV), followed by the East Bay (Station II) and West Bay (Stations I and V) (Figure 2A). HF183 (average concentration = 1.91 log_10_ copies/mL) was detected in six out of nine stations, making it the most frequently detected marker, covering mostly the west area of the lake (Stations I, V, XV, and XVI). However, this does not necessarily mean that it had the highest copy number. On the other hand, DuckBac (average concentration = 1.32 log_10_ copies/mL) was detected in four sites while BoBac was not detected in any of the study sites throughout the sampling duration.

### 3.3. Seasonal Patterns in the Abundance of Bacteroides MST Markers

**Figure 3:**
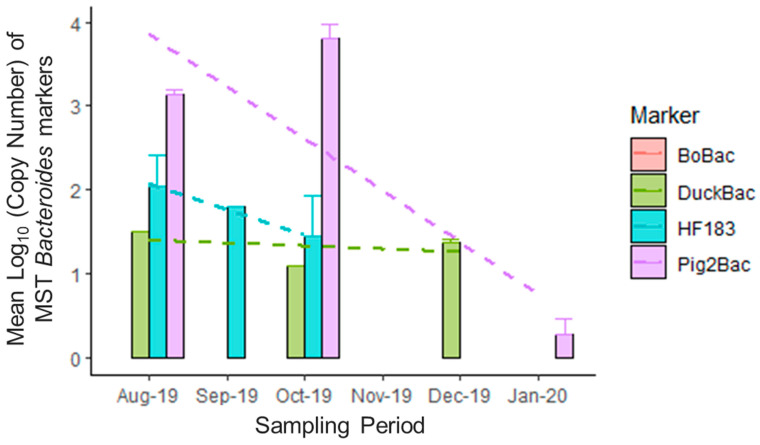
**Temporal trend of mean marker abundance across Laguna Lake monitoring stations from August 2019 to January 2020.** The mean copy number of each marker across all sites was obtained to determine the temporal trend of fecal contamination in the given sampling period. The period of August to October 2019 represents the wet season, while November 2019 to January 2020 represents the dry season. The broken lines represent the general trend or pattern of abundance of each marker throughout the 6 months of the sampling period.

**Figure 4:**
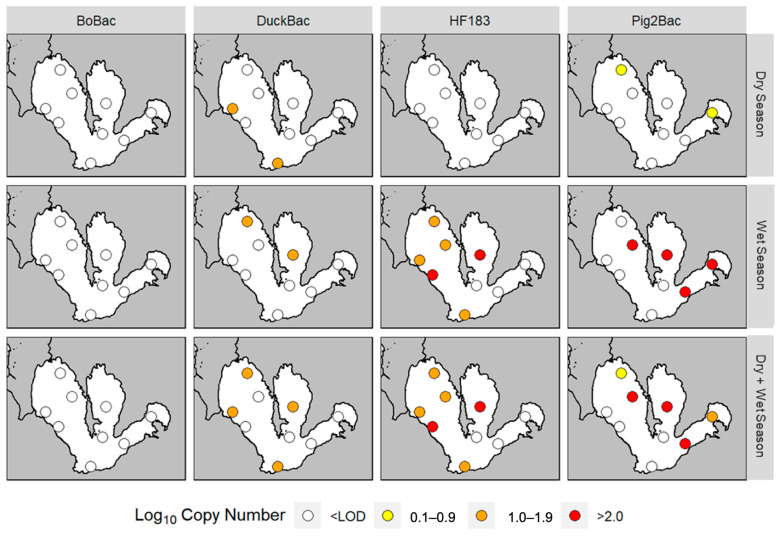
**Seasonal patterns and spatial distribution in terms of the abundance of different MST *Bacteroides* markers (log_10_ copy number mL^−1^) during the wet and dry seasons, and both**. To visualize the seasonal patterns of fecal contamination for each source, the mean copy number of each marker across all sites was obtained and plotted against the map of the lake generated through R programming. The period of August to October 2019 represents the wet season, while November 2019 to January 2020 represents the dry season.

Higher marker concentrations were notable during the months of August, September, and October 2019 (Figure 3). Furthermore, the levels of DuckBac were generally constant, as observed from August to December 2019, except that it was not detected during November 2019. On the other hand, traces of Pig-2-Bac were detected in January 2020. Figure 4 shows the seasonal pattern of marker abundance across sampling sites. Marker concentrations from all sampling sites combined were higher during the wet season compared to the dry season (Figure 4). The spatial characteristics can also be considered as a major factor in the distribution and occurrence of the markers used.

### 3.4. Correlation between Bacteroides Markers, Fecal Coliforms, and Water Quality Parameters

The correlations between the monthly abundance (August 2019 to January 2020) of fecal contamination from each host source and environmental parameters (fecal coliform, BOD, DO, pH, ammonia, nitrate, and phosphate) were tested (Table 4). The abundance of DuckBac and Pig-2-Bac was not significantly correlated with any of the environmental variables, while the abundance of HF183 was significantly correlated with levels of phosphate present in the lake (ρ = 0.45; *p* < 0.001).

## 4. Discussion

**Validation of *Bacteroides* 16S rDNA-based markers for MST.** The human marker HF183, initially developed by Bernhard and Field [8] from the 16S rRNA gene of *Bacteroides dorei* and closely related taxa, has been widely used for two decades and is well characterized with regard to host-sensitivity and -specificity [27]. The improved primer pair HF183/BacR287 [10] was evaluated in this study, and relatively high values for sensitivity (0.88) and specificity (0.99) were obtained. The lower sensitivity of HF183 corroborated previous reports, which stated that this marker was more prevalent in composite water samples (raw, septic, and effluent wastewater) than in individual human fecal samples [28]. Meanwhile, the specificity value obtained in this study (0.99) was higher than the previously reported overall host-specificity value (0.946) [27]. The occasional presence of the HF183 marker in nonhuman fecal samples, including samples from chickens, has been previously reported in other validation studies [29,30]. Because chicken fecal samples were collected from backyard farms where animals are exposed to human contact, the possibility of cross-contamination between humans and chickens cannot be ruled out. The uptake of HF183 can also occur if chickens drink human wastewater [29].

The high host-specificity and -sensitivity of the Pig-2-Bac marker designed by Mieszkin [5] and Gourmelon [23] from pig feces and slurries make it ideal for detecting pig fecal contamination. In this presence/absence evaluation, no nontarget fecal sample was positive in the Pig-2-Bac assay. This was consistent with the previous validation studies [6,23] [31], which applied more sensitive qPCR platforms. This result suggests that the marker performs well even in endpoint PCR for qualitative assessment and is ideal for use in MST.

Because avian feces are potential sources of pathogens, genetic markers have been proposed for duck, goose, crane, and poultry feces, but most of these markers are not based on the *Bacteroides* 16S rRNA gene [32,33,34]. To date, PCR assays targeting duck and chicken markers have not been well established. Kobayashi [12] designed three markers (Chicken-Bac, Duck-Bac, and Chicken/Duck-Bac) for the detection and estimation of chicken and duck feces in the environment. We found that DuckBac performed better (S = 0.94; R = 1.00) than reported by Kobayashi (S = 0.85; R = 0.96) [12] and ChickenBac had a poorer performance (S = 0.65; R = 0.31) than that reported by Kobayashi [12] (S = 0.70; R = 0.92). In the current study, the ChickenBac marker was observed in fecal samples from ducks, pigs, and cows, confirming the presence of this marker in animals other than chickens. Hence, only the Duck-Bac marker was used in this study because the ChickenBac marker is not recommended for source tracking due to its low sensitivity and specificity.

In the estimation of contamination from cattle, BoBac performed poorly (R = 0.10) because of an extreme cross-reaction with all nontarget host groups. This result was not consistent with other studies, which have reported the BoBac marker to be 0.93–0.97 specific (11, 22) with only a few cross-reactions with human and dog fecal samples. This occurrence might have resulted from factors such as diet and cohabitation, which can influence the sharing of host-associated microorganisms among animals [35]. Because our results were based on presence/absence data, which are qualitative estimates, conclusive findings regarding the source of contamination based on this marker may not be accurate. Given that the specificity and distribution of a marker in host populations can vary in terms of geographical and temporal aspects, this type of data is not sufficient to establish the suitability and accuracy of the markers for successful field application [28]. The sensitivity and specificity values of the host-associated markers reported in this study prove that there is a need to validate these markers before conducting quantitative MST studies in other geographical regions [36]. Furthermore, a high degree of variability in microbes in the gut occurs due to cohabitation, diet, physiology, feeding habits, and geographical stability [37].

**Quantification and spatial distribution of host-associated *Bacteroides* markers in lake water samples.** Human contamination was frequently detected in the west area of the lake (Stations I, V, XV, and XVI) (Figure 4). These findings align with the most recent land cover data of the Laguna Lake basin, the area surrounding the lake. Based on the 2015 land cover map of the basin [38], built-up areas on the northwest to the west side of the lake are prevalent. These areas, in Metro Manila and the Laguna Province, have been converted, mostly by private owners for industrial or commercial use, but also for residential purposes [39]. Urban sprawl has led to an increase in the amount of domestic waste being disposed into local waterways [40]. These wastes are produced from human activities such as bathing, laundry, cleaning, cooking, and washing [41]. As may be inferred, domestic waste is a major contributor to pollution in Laguna Lake. This is also reported in the Pilot Ecosystem Account for Laguna de Bay Basin [4], which stated that 81% of BOD came from domestic wastes. De la Peña et al. [42] also reported that sewage was the most dominant contributor to fecal contamination in Laguna Lake. This could be linked to a lack of sewerage systems in the area, which transport sewage from its source to a treatment and disposal facility [41]. In Metro Manila, only around 12% of households are connected to a sewer line [43]. This problem of sewerage systems, as well as sewage and septage management and treatment, should be addressed.

Pig fecal contamination was most abundant in the Central Bay (Station IV), followed by the East Bay (Station II) and West Bay (Stations I and V). The high abundance of pig fecal contamination in these areas can be attributed to the prevalence of swine industries in these regions. According to the Selected Statistics on Agriculture report [44], the swine industry accounts for 82% of the total value of production in the livestock group in the Philippines. In 2019, the total swine population was 12.71 million heads. In terms of regional distribution, Calabarzon, where Laguna Lake is located, accounted for 16% of the total swine inventory. 

It is important to note that the swine population is made up of both backyard and commercial farming operations. Backyard farms (<20 heads) account for around 64% of the swine population in the Philippines, and commercial farms (>1000 heads) account for the remaining 36% [44]. Waste disposal monitoring by the Environmental Management Bureau only applies to farms that produce more than 30 m^3^ of wastewater discharge per day [45]. This may suggest that backyard and small farms (1–999 swine heads) are likely contributors to swine fecal contamination in the Central and East Bays due to the discharge of waste products into nearby creeks and rivers [46], which could later drain into the lake. Meanwhile, large commercial farms are mainly established near Metro Manila to meet the demand for meat products in this area [47]. The foregoing facts suggest that pig fecal contamination in the West Bay could be associated with commercial farming. 

In addition to pig fecal contamination, duck fecal contamination was confirmed in four of the nine lake sites: the Central, West, South, and San Pedro Bays. Like the swine industry, the poultry industry in the Philippines is also composed of commercial (>100 birds) and backyard operations. Backyard poultry farms are located almost anywhere near the markets and can operate in densely populated areas as long as the birds are caged, while bigger farms are located in less urbanized areas [48]. Compared to the chicken population, which comprises 186 million heads, the duck population comprises only 11.58 million heads according to the inventory made by the Philippine Statistics Authority in 2019 [44]. Among the different administrative regions, Calabarzon is among the highest producers of poultry in terms of percentage distribution. The presence of duck feces in the lake sites may be attributed to the duck-raising industries located along the lake shoreline. Because the lake provides an abundant water supply and freshwater snails that serve as food for ducks, it has become a common breeding site for ducks [49]. The largest duck-raising farm in the province of Laguna is in the town of Victoria [49], close to the South Bay station, where duck-associated markers were detected. 

As for the cow fecal marker, non-detection may have been a result of significant degradation or dilution of the marker during in-stream transport [50]. The marker concentration in the lake could be very low to begin with, for its successful detection in the samples. The non-detection of the BoBac marker suggests that cattle feces is unlikely to be a major contributor of contamination at the sampling sites. This is consistent with the recent MST data derived from a library-dependent method (rep-PCR), where only 3.31% (11/332) of the environmental samples were reported to be contaminated with cattle fecal matter [42]. However, our finding differs from that of Abello et al. [51], which revealed cattle-associated contamination in the West Bay (Stations I and V) using the heat-labile *E. coli* toxin gene marker LTIIA. The differences in these results demonstrate the distinct properties and functions of different MST methods. Because MST is an emerging method, a “toolbox” approach, i.e., a combination of different strategies, is recommended for the definitive identification of pollutant sources and the optimal accuracy of data interpretation [7]. 

**Seasonal patterns in the abundance of *Bacteroides* MST markers.** The Laguna Lake watershed has two climate types based on Coronas’ modified climate classification system. The majority of the surrounding region falls under the Type I classification, exhibiting periods of low rainfall (dry season) during November to April, and high levels of precipitation (wet season) between May and October [52,53]. The higher marker concentrations observed from August to October in this study can be explained by higher levels or more frequent precipitation during these months, which may facilitate the movement of fecal matters from point sources. Seasonal changes can influence marker concentrations through the entry of fecal contaminants via various pathways during heavy rainfall events [54]. 

Laguna Lake has around 100 rivers and streams that drain into the lake, 22 of which are major river tributaries [55] that contribute to around 70% of the total lake inflow [56]. Fecal contamination, which comes from sewage, discharges from wastewater treatment plant channels, or the direct defecation of livestock, wildlife, and domestic animals [54,57], enters the lake during heavy rainfall via storm water runoff. Previous studies have shown that periods of intense precipitation and high river discharge are associated with an exponential increase in the concentration of FIB in water bodies [58,59]. FIB densities in rivers are typically 10 to 1000 times higher during flood events compared to during dry periods, depending on storm severity and watershed characteristics [60]. Conversely, increased precipitation can also reduce the pathogen concentration in surface water due to dilution [14,61]. In Laguna Lake, rainfall-related seasonal variations were found to influence water quality [40]. Rainfall affects the turbidity and pollution of water due to inflows from domestic waste or industrial effluents [62]. The absence or low detection of markers in most of the samples during the dry season indicated low levels of fecal pollution. During dry weather events, the increase in temperature can cause pathogens to die off, thereby lowering their concentration in the environment [63]. Sunlight raises the level of reactive oxygen species in the environment, which can affect *Bacteroidales* species depending on their oxygen protection mechanisms [64]. Furthermore, because streamflow is mainly influenced by precipitation runoff in the watershed [65], decreased streamflow during the dry season is likely to reduce the water inflow in the watershed. Thus, even though river tributaries were heavily contaminated with fecal coliforms from November 2019 [66] to January 2020 [67], these contaminants were detected at only low levels in the representative samples. 

**Correlation between *Bacteroides* markers, fecal coliforms, and water quality parameters.** Among the markers, only the concentration of the human-associated marker appeared to have a significant correlation with the level of phosphate recorded in the lake. Phosphorus is present in inorganic and organic forms in natural waters [68]. It is also found in feces and waste materials, synthetic detergents, and household cleaning products [69]. It is present in sewage mainly as orthophosphate, and its removal is essential during sewage treatment [70]. Phosphorus and nitrogen promote algal growth and degrade water quality [71]. Therefore, limiting phosphorus discharge from wastewater treatment plants is a critical component in preventing the eutrophication of receiving waters [72].

The significant correlation between the level of phosphate and the prevalence of HF183 reflects the lack of connection to sewerage systems as well as the presence or level of wastewater treatment [73]. Issues in sewage management and treatment should be addressed immediately to efficiently reduce sewage contamination, effectively remove phosphorus in wastewaters, and prevent further eutrophication of the lake.

## 5. Conclusions

The validation of the *Bacteroides* 16S rDNA markers revealed the sensitivity and specificity of the three host-associated markers. HF183, Pig-2-Bac, and DuckBac were efficient *Bacteroides* markers for detecting fecal contamination from humans, pigs, and ducks, respectively. This study established that these three markers can be utilized with confidence in MST studies in Laguna Lake. Additionally, their performances are considered useful for assessing fecal contamination in other water bodies, especially those in close proximity to the lake. However, we recommend the validation of the markers prior to their use for quantitative MST studies in a new region in order to establish the marker performance.

The higher marker concentrations during the wet season compared to the dry season suggested that seasonal factors, such as precipitation, influence the occurrence and abundance of markers in lake water. Among the water quality parameters (fecal coliform, DO, BOD, pH, nitrate, ammonia, and phosphate level), only the phosphate level showed a significant correlation with human-associated markers.

The findings of this study can support policymakers in enforcing strict regulations on proper waste management and disposal in the areas surrounding Laguna Lake. Specifically, the data on the abundance of pig-associated contamination may guide environmental sectors in considering the monitoring of backyard and small commercial farms to mitigate pollution. Additionally, the issues regarding the limited sewerage systems should be addressed to reduce human- or sewage-derived contamination. These strategies will help in preserving lake water quality and preventing further degradation of the water body.

## Figures and Tables

**Table 1:** Primer sequences of host-associated *Bacteroides* 16S rDNA markers.

Assay	Primer Name	Sequences (5′-3′)	Amplicon Size (bp)	Annealing Temp (°C)	Reference
Cow	BoBac367 F	GAAG(G/A)CTGAACCAGCCAAGTA	100	55	[11]
	BoBac367 R	GCTTATTCATACGGTACATACAAG			
Pig	Pig-2-Bac41F	GCATGAATTTAGCTTGCTAAATTTGAT	116	55	[6]
	Pig-2-Bac163R	ACCTCATACGGTATTAATCCGC			
Human	HF183F	ATCATGAGTTCACATGTCCG	126	58	[10]
	BacR287	CTTCCTCTCAGAACCCCTATCC			
Duck	Bac366F	TTGGTCAATGGGCGGAAG	108	60	[12]
	Duck474R	GCACATTCCCACACGTGAGA			
Chicken	C160F	AAGGGAGATTAATACCCGATGATG	105	60	[12]
	Bac265R	CCGTTACCCCGCCTACTAC			

**Table 2:** Number and percentage of positive samples in each host-specific assay.

Host	HF183 (Human)	BoBac (Cow)	Pig-2-Bac (Pig)	DuckBac (Duck)	ChickenBac (Chicken)
Human	21/24 (88)	17/20 (85)	0/20 (0)	0/20 (0)	0/20 (0)
Cow	0/20 (0)	18/20 (90)	0/20 (0)	0/20 (0)	15/20 (75)
Pig	0/15 (0)	12/15 (80)	15/15 (100)	0/15 (0)	15/15 (100)
Duck	0/18 (0)	15/15 (100)	0/18 (0)	17/18 (94)	15/15 (100)
Chicken	1/20 (5)	19/20 (95)	0/20 (0)	0/20 (0)	15/23 (65)

Note: Corresponding percentages are enclosed in parentheses.

**Table 3:** Performance characteristics of *Bacteroides* 16S rDNA markers.

Marker	Sensitivity	Specificity	Accuracy	Positive Predictive Value	Negative Predictive Value
Pig-2-Bac	1.00	1.00	1.00	1.00	1.00
DuckBac	0.94	1.00	0.99	1.00	0.99
HF183	0.88	0.99	0.96	0.95	0.96
BoBac	0.90	0.10	0.28	0.22	0.78
ChickenBac	0.65	0.31	0.40	0.25	0.61

**Table 4:** Spearman correlation (ρ) of *Bacteroides* marker concentration with environmental parameters. The measurements of environmental parameters were obtained from the Laguna Lake Development Authority (LLDA) and covered the period of August 2019 to January 2020.

Environmental Parameter	Correlation (ρ) with *Bacteroides* Marker Concentration (log_10_ Copy Number mL^−1^)		
	DuckBac	Pig-2-Bac	HF183
Ammonia (mg/L)	−0.08	0.07	0.27
BOD (mg/L)	−0.21	−0.23	0.05
DO (mg/L)	−0.09	0.04	−0.25
Fecal coliform (MPN/100 mL geomean)	0.20	−0.02	0.06
Nitrate (mg/L)	0.14	−0.08	−0.22
pH (units)	0.02	0.07	−0.03
Phosphate (mg/L)	0.12	0.01	0.45 **

** *p* < 0.001.

## Data Availability

All relevant data are included in this manuscript.
